# Iron‐Deficiency Anemia Elevates Risk of Diabetic Kidney Disease in Type 2 Diabetes Mellitus

**DOI:** 10.1111/1753-0407.70060

**Published:** 2025-02-19

**Authors:** Bin Huang, Wenjie Wen, Shandong Ye

**Affiliations:** ^1^ Department of Endocrinology The First Affiliated Hospital of USTC, Division of Life Science and Medicine, University of Science and Technology of China Hefei Anhui China; ^2^ Anhui Province Engineering Research Center for Dental Materials and Application, School of Stomatology Wannan Medical College Wuhu China

**Keywords:** diabetic kidney disease, iron metabolism, iron supplementation, iron‐deficiency anemia, Mendelian randomization

## Abstract

**Objective:**

This study aims to explore the link between iron deficiency anemia (IDA) and diabetic kidney disease (DKD) and assess the safety of iron supplementation. It also investigates key mechanisms and molecules involved in iron deficiency's role in disease development.

**Methods:**

A retrospective analysis was conducted on 1,398 T2DM patients using propensity score matching to identify risk factors for DKD. Mendelian randomization (MR) was used to explore causal relationships between IDA, iron supplementation, liver iron content, and DKD. The GSE27999 dataset was analyzed to examine how an iron‐deficient diet affects kidney‐related gene expression. Key pathways and molecules were identified through GSEA, GO/KEGG, and PPI analysis.

**Results:**

Retrospective data showed a correlation between hemoglobin levels and DKD risk. Logistic regression confirmed that IDA increased DKD risk independently of other factors. MR revealed a causal link between IDA and DKD, with no significant effect from iron supplementation. GSE27999 analysis identified 580 differentially expressed genes, enriched in pathways like cytokine signaling, oxidative biology, and small molecule transport. PPI analysis highlighted 10 key hub genes, including Cyp2d26 and Fgf4.

**Conclusion:**

IDA increases susceptibility to DKD, possibly through oxidative stress and altered small molecule transport. However, iron supplementation does not appear to increase the risk of DKD.

## Graphical abstract

The chronic inflammation in diabetes often leads to functional iron deficiency, which in turn results in iron‐deficiency anemia (IDA). Abnormal iron metabolism and insufficient oxygen supply caused by anemia both play a role in kidney damage. However, there is currently limited research on the association between IDA and diabetic kidney disease (DKD) risk. To examine the causal connection between IDA and the development of DKD, alongside assessing the safety of iron supplementation therapy. Furthermore, the study also aims to investigate potential mechanisms and essential molecules involved in this pathogenesis. Our research has found that IDA can elevate the risk of developing DKD. Kidney damage arising from iron deficiency may be connected to oxidative stress, heightened transport of small molecules, and additional factors. Administering iron supplementation as a remedy for IDA poses no risk concerning the occurrence of DKD, and an augmentation in liver iron content is linked to a diminished risk of DKD.

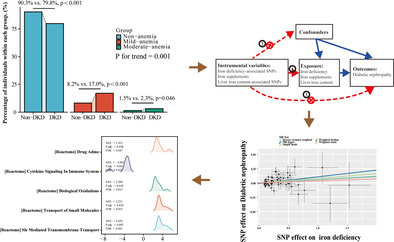

**Summary**

- This study reveals that iron‐deficiency anemia (IDA) significantly increases the risk of developing diabetic kidney disease (DKD).
- Iron supplementation therapy for IDA in diabetic patients does not raise the risk of DKD, and increased liver iron content may reduce DKD risk.

## 1 Introduction

Iron stands as an indispensable mineral necessary for a multitude of molecules to uphold their innate structure and function [1]. It plays a pivotal role in ensuring the survival, growth, and proliferation of cells. Iron deficiency stands as the most prevalent shortfall in micronutrients, impacting almost a third of the populace and emerging as the prime catalyst for global anemia [2]. Given the absence of a mechanism for the body to expel iron, barring blood loss or cellular renewal, systemic iron levels remain under stringent regulation [3]. Either deficiency or overload will contribute certain pathogenic outcomes [4, 5, 6].

Diabetic kidney disease (DKD) stands as one of the most prevalent and detrimental chronic complications stemming from diabetes [7]. The pathogenesis of DKD is intricate and, despite thorough research, remains not fully comprehended. In recent years, diverse animal models of DKD have exhibited instances of iron overload and anomalous deposition of iron within the kidneys [8]. Ferroptosis, a novel form of programmed cell death, has progressively come to light as a pivotal player in the onset and progression of DKD [9, 10]. Notably, renal tubular injury, particularly the impairment of proximal tubular cells (PTC), occupies a vital role in the initiation and advancement of DKD [11]. PTC play a pivotal role in renal reabsorption mechanisms, necessitating considerable ATP consumption. Consequently, they possess a profusion of mitochondria to cater to the heightened metabolic activity and energy requisites of the tissue [12]. On one hand, iron‐deficiency anemia (IDA) leads to an escalated oxygen consumption in PTC due to the heightened demands for iron reabsorption in renal tubules. Meanwhile, the insufficient oxygen supply brought about by diabetes and anemia could conceivably yield disruptions in iron metabolism and oxidative stress‐induced harm within PTC. As renal function declines, it can lead to inadequate production of erythropoietin and abnormal iron ion reabsorption, thereby increasing the risk of IDA. This has become a consensus. However, whether IDA can increase the susceptibility to DKD in individuals with normal estimated glomerular filtration rate (eGFR) is currently an area lacking relevant research.

Mendelian randomization (MR) is an epidemiological analytical approach that enhances causal inference. The MR design employs genetic variants as instrumental variables (IVs) for the target exposure, typically single nucleotide polymorphisms (SNPs), which are randomly dispersed and remain unaffected by environmental influences and other potential confounding factors [13]. Consequently, the MR design offers a robust method to accurately elucidate causal associations within intricate disorders. This study is designed to examine the causal connection between IDA and the development of DKD in normal eGFR, alongside assessing the safety of iron supplementation therapy by utilizing retrospective clinical data and MR analysis. Moreover, the study also aims to scrutinize potential pathogenic mechanisms and essential molecules employing bioinformatics analysis. This endeavor promises to unveil fresh perspectives on comprehending the impact of disrupted iron metabolism on the progression of DKD and introduces innovative avenues for forthcoming therapeutic strategies.

## 2 Materials and Methods

### 2.1 Participants of Clinical

A total of 669 individuals with T2DM and DKD who were admitted to the Department of Endocrinology at the First Affiliated Hospital (Anhui Provincial Hospital) of the University of Science and Technology of China between July 2015 and September 2022 were included in this study. An additional 669 T2DM patients without DKD formed the control group, and they were matched for age, sex, and BMI using propensity score matching (PSM). Informed consent requirements were waived since this was a retrospective analysis of data extracted from participants' medical records. The diagnosis of DKD was established based on two instances of elevated urinary albumin‐to‐creatinine ratio (UACR), one at the time of hospital admission and another during a follow‐up outpatient visit 3 months after discharge, both exceeding 30 mg/g. Exclusion criteria encompassed: (1) Type 1 diabetes mellitus (T1DM) or acute diabetes‐related complications; (2) Ongoing iron supplement treatment; (3) Presence of conditions like cancer, severe liver disease, chronic kidney disease (CKD) defined as eGFR ≤90 mL/min/1.73 m^2^, or other co‐existing factors such as pregnancy or rheumatic connective tissue disease; (4) Severe anemia with hemoglobin levels below 60 g/L; and (5) Other kidney diseases potentially impacting renal function and/or proteinuria, such as urinary tract infections. Patient data, including diabetes duration, age, gender, body mass index (BMI, kg/m^2^), and medication usage (insulin, anti‐diabetes drugs, and RAAS inhibitors continuously taken for at least 3 months) were extracted from medical records. Due to the increased risk of false positives for UACR associated with high glucose, we conducted two UACR tests during the hospitalization: one within 1–2 days after admission and another within 1–2 days before discharge. The results of the second test, which reflected stable glucose control, were used for this study. All patients underwent diabetes assessment (HbA1c), routine blood tests (hemoglobin), biochemical assessments (ALT, AST, TBIL, Cr, and uric acid levels), lipid profile analysis (TG, LDL‐C, HDL‐C, and TC levels), and baseline ferritin levels. eGFR was calculated using the formula:194×Cr ^−1.094^ $\times {\mathrm{Age}}^{-0.287}\;\left(\times 0.739\;\text{for female patients}\right)$. The diagnosis of IDA aligns with the following criteria [14]: (1) Presence of evident iron deficiency etiology and corresponding clinical symptoms (such as fatigue, dizziness, palpitations, etc.); (2) Microcytic hypochromic anemia presentation: Hemoglobin (Hb) levels below 120 g/L in males and below 110 g/L in females; Mean Corpuscular Volume (MCV) less than 80 fl, Mean Corpuscular Hemoglobin (MCH) less than 27 pg., Mean Corpuscular Hemoglobin Concentration (MCHC) less than 0.32. The grading criteria for anemia are as follows: Hemoglobin levels ranging from 90 to 120 g/L (90–110 g/L for females) correspond to mild anemia; levels between 60 and 90 g/L denote moderate anemia.

### 2.2 MR Design and Data Sources Description

**FIGURE 1:**
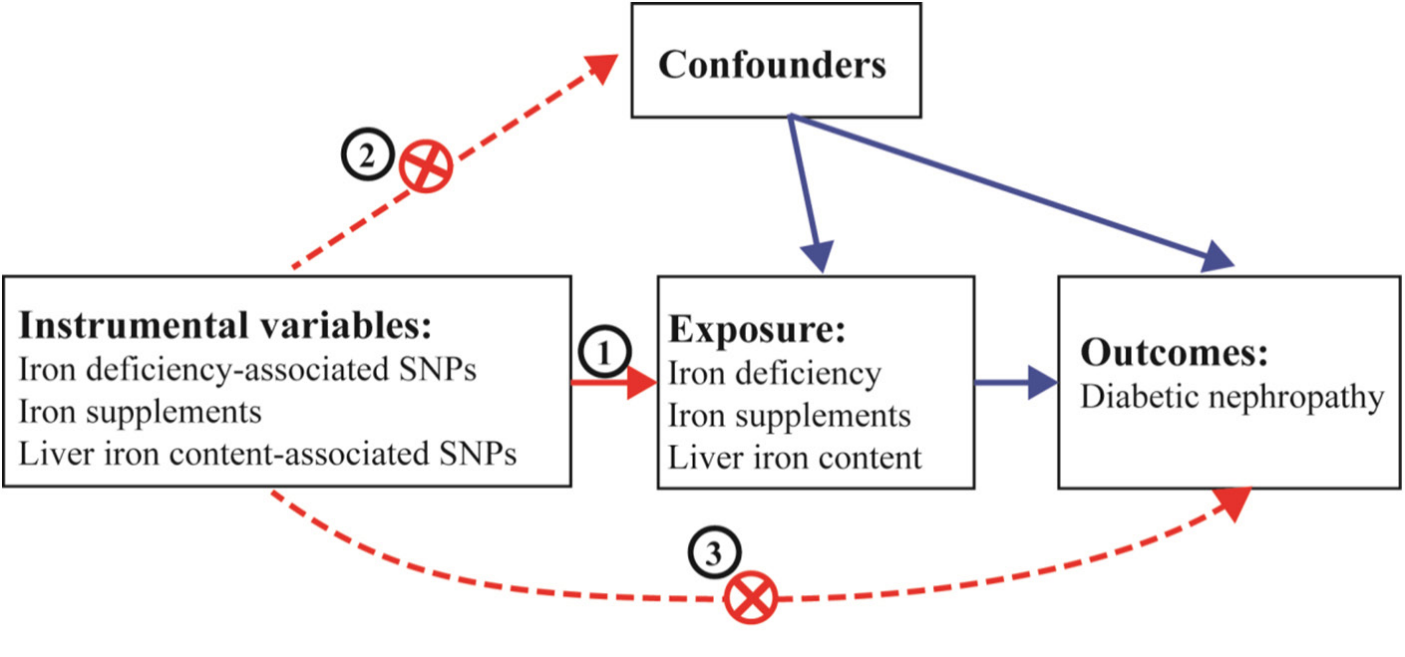
The framework of the current MR study.

The framework of the present MR study is depicted in Figure 1. In this study, we employed genetic variants as IVs for the MR analysis. The credibility of our MR study rested on three fundamental assumptions [15]: [1] relevance assumption: the genetic variants exhibit a robust connection with the exposure; [2] independence assumption: the genetic variants are not linked to any confounding factors that might act as mediators between the exposure and the outcome; and [3] exclusion‐restriction assumption: the genetic variants solely influence the outcome through the exposure. Concurrently, these SNPs were chosen based on a significance threshold of *p* < 5 × 10^−5^ and a minor allele frequency exceeding 0.01. Furthermore, we computed the *F* statistics of the SNPs to explore the potential presence of weak instrument bias. The genetic association concerning DKD was derived from a study involving 3283 European cases and 181 704 European controls. The summary data for Genome‐Wide Association Studies (GWAS) were sourced from public databases curated by the European Bioinformatics Institute (EBI) available at https://gwas.mrcieu.ac.uk/datasets/finn‐b‐DM_NEPHROPATHY_EXMORE/. Additionally, the summary data for GWAS on iron deficiency (https://gwas.mrcieu.ac.uk/datasets/finn‐b‐D3_ANAEMIA_IRONDEF_NAS/), liver iron content (https://gwas.mrcieu.ac.uk/datasets/ebi‐a‐GCST90016674/), and iron supplement usage (https://gwas.mrcieu.ac.uk/datasets/ukb‐b‐14863/) were procured from public databases also housed within the EBI. The Ethics Committees corresponding to each GWAS had granted approval for their respective studies.

### 2.3 GSE27999 Analysis

We downloaded the dataset GSE27999 from GEO (http://www.ncbi.nlm.nih.gov/geo). In the dataset, samples were divided into two groups (normal diet and iron deficient diet group). After consolidation and normalization of the RNA‐seq data, 580 DEGs involved in iron deficiency kidney were screened by the limma package (*p* < 0.05, |logFC| ≥ 2). To understand the biological function and signaling pathways of the commonly shared DEGs involved in kidney of iron‐deficient diet, the 580 identified DEGs were subjected to enrichment analysis within the Gene Ontology [16] (GO; http://www.geneontology.org/) database and pathway analysis within the Kyoto Encyclopedia of Genes and Genomes [17] (KEGG; https://www.kegg.jp/) using the R package ClusterProfile (Adjusted *p* < 0.05 and *Q* < 0.05). Gene set enrichment analysis (GSEA) [18] (http://software.broadinstitute.org/gsea/index.jsp) is a kind of gene expression data based on molecular signatures database method. After 1000 permutation tests, the most significant pathways related to iron deficient DEGs in kidney were all selected as the critical *p* value of significance level, the false discovery rate *Q* value and the family wise error rate *p* value. STRING [19] (Search Tool for the Retrieval of Interacting Genes) (https://string‐db.org/cgi/input.pl) is an online tool designed to evaluate the protein–protein interaction (PPI) network. Cytoscape is an open source software project for integrating biomolecular interaction networks with high‐throughput expression data and other molecular states into a unified conceptual framework. CytoHubba is a Cytoscape plugin, was employed to discover the top 10 hub genes in the network.

### 2.4 Statistical Analysis

IBM SPSS Statistics version 22.0 (IBM Co., Armonk, NY, USA) was employed for the analysis. The missing values in this study are missing completely at random, and as the univariate missing rate is less than 10%, they were handled by multiple imputation before the analysis. A two‐tailed *p* value of less than 0.05 was considered statistically significant. Descriptive statistics for continuous measurements were provided as the mean (SD) or the median (IQR), depending on their distribution. Categorical variables were described using frequencies (percentages). To compare the two groups of patients, we utilized *T* tests, chi‐square tests, or Mann–Whitney *U* tests. For analyzing the risk factors for DKD, a logistic regression model was used to estimate the odds ratio (OR) with a 95% confidence interval, while adjusting for potential confounding variables. To explore the relationship between hemoglobin levels and UACR, we employed Spearman's correlation analysis. The effect of iron‐related markers on DKD was estimated using the inverse variance‐weighted (IVW) method [20]. Additionally, the weighted‐median method served as a supplementary approach to IVW. Based on the outcome of heterogeneity, the random effects model IVW was selected simultaneously. Heterogeneity was evaluated using Cochrane's *Q* value. We assessed the horizontal pleiotropy of SNPs using both the MR‐Egger intercept and MR‐PRESSO methods. Notably, the outlier test within MR‐PRESSO addressed horizontal pleiotropy by removing outliers. For MR analysis, the R package “TwoSampleMR” was utilized. MR‐PRESSO was executed using the R package “MRPRESSO”. The relationship between SNPs and phenotype was examined through “PhenoScanner V2”. All statistical analyses of GSE27999 were conducted using R software version 4.3.1. Visual plots were generated using GraphPad Prism (version 8.0; GraphPad Software, La Jolla, CA).

## 3 Results

### 3.1 Demographic and Metabolic Characteristics of Study Subjects

The data of 1398 patients with T2DM (55.72% male) were evaluated. The mean age of the study subjects was 56.86 ± 14.98 years, ranging from 19 to 84 years. The duration of diabetes ranged from 0 to 41 years. Patients in the DKD group had significantly higher UACR, HBA1C, FPG, ALP, UA and TG levels but a lower fasting C‐peptide, TBIL, eGFR, Hb levels than those in the non‐DKD group (all *p* < 0.05). There were no significant differences in sex, age, BMI, history of smoking, history of drinking, drug usage, and so on between the two groups (all *p* > 0.05) (Table 1).

**TABLE 1:** Demographic and metabolism characterization of study subjects.

Characteristics	Non‐DKD group (*N* = 669)	DKD group (*N* = 669)	*p*
General data			
Gender (male/female)	383/386	396/273	0.471
Age (year)	56.87 ± 14.78	56.86 ± 15.16	0.994
BMI (kg/m^2^)	24.97 ± 3.88	25.14 ± 4.28	0.443
History of smoking (%)	61 (9.1%)	72 (10.8%)	0.315
History of drinking (%)	79 (11.8%)	92 (13.8%)	0.287
Comorbidity or complication			
HBP (%)	176 (26.3%)	194 (29.0%)	0.271
ASCVD (%)	259 (38.7%)	293 (43.8%)	0.059
Drug usage			
Insulin (%)	102 (15.2%)	115 (17.2%)	0.335
Metformin (%)	376 (56.2%)	369 (55.2%)	0.700
GLP‐1R (%)	67 (10%)	54 (8.1%)	0.215
SGLT2i (%)	132 (19.7%)	151 (22.6%)	0.203
DPP‐4i (%)	157 (23.5%)	129 (19.3%)	0.062
RAASi (%)	78 (11.7%)	91 (13.6%)	0.285
Glucose metabolism			
Duration of diabetes (year)	8 (5, 17)	7 (3, 16)	0.247
HbA1c (%)	8.50 ± 2.18	9.11 ± 2.21	**< 0.001**
FPG (mmol/L)	9.00 ± 5.18	10.75 ± 6.43	**< 0.001**
Fasting C‐peptide(nmol/L)	0.61 (0.3675, 1.13)	0.53 (0.32, 0.96)	**0.013**
UACR (mg/gCr)	13.89 (9.54, 19.74)	82.5 (44.26, 342.09)	**< 0.001**
Biochemical data			
ALT (IU/L)	18.65 (13.375, 28.225)	18.5 (12.53, 28)	0.567
AST (IU/L)	19.3 (16, 24.575)	19 (15.5, 25.7)	0.615
ALP (μmol/L)	76 (63, 93)	80 (67, 101)	**< 0.001**
TBIL (μmol/L)	12.7 (10.1, 16.6)	11.7 (8.7, 15.1)	**< 0.001**
DBIL(μmol/L)	2.6 (2, 3.5)	2.4 (1.8, 3.425)	0.079
eGFR (mL/min/1.73 m^2^)	116.03 (106.64, 137.09)	109.11 (96.04, 135,79)	**0.032**
Uric acid (umol/L)	317.81 ± 91.39	332.91 ± 105.26	**0.005**
Hb (g/L)	132.67 ± 17.05	129.35 ± 19.56	**0.001**
Ferritin (ng/mL)	131.90 (66.40, 221.85)	125.15 (58.08, 226.91)	0.597
Lipid profile			
TG (mmol/L)	1.49 (1.03, 2.17)	1.65 (1.1, 2.555)	**0.002**
TC (mmol/L)	4.5 (3.83, 5.2)	4.53 (3.7075, 5.34)	0.922
LDL‐c (mmol/L)	2.53 (2.01, 3.05)	2.51 (1.9475, 3.09)	0.433
HDL‐c (mmol/L)	1.04 (0.88, 1.21)	1.02 (0.87, 1.2)	0.209

*Note:* Data are *n* (%), mean ± SD, or median (interquartile range).

### 3.2 Hemoglobin Concentration and the Degree of Anemia Are Related to the Incidence of DKD

**FIGURE 2:**
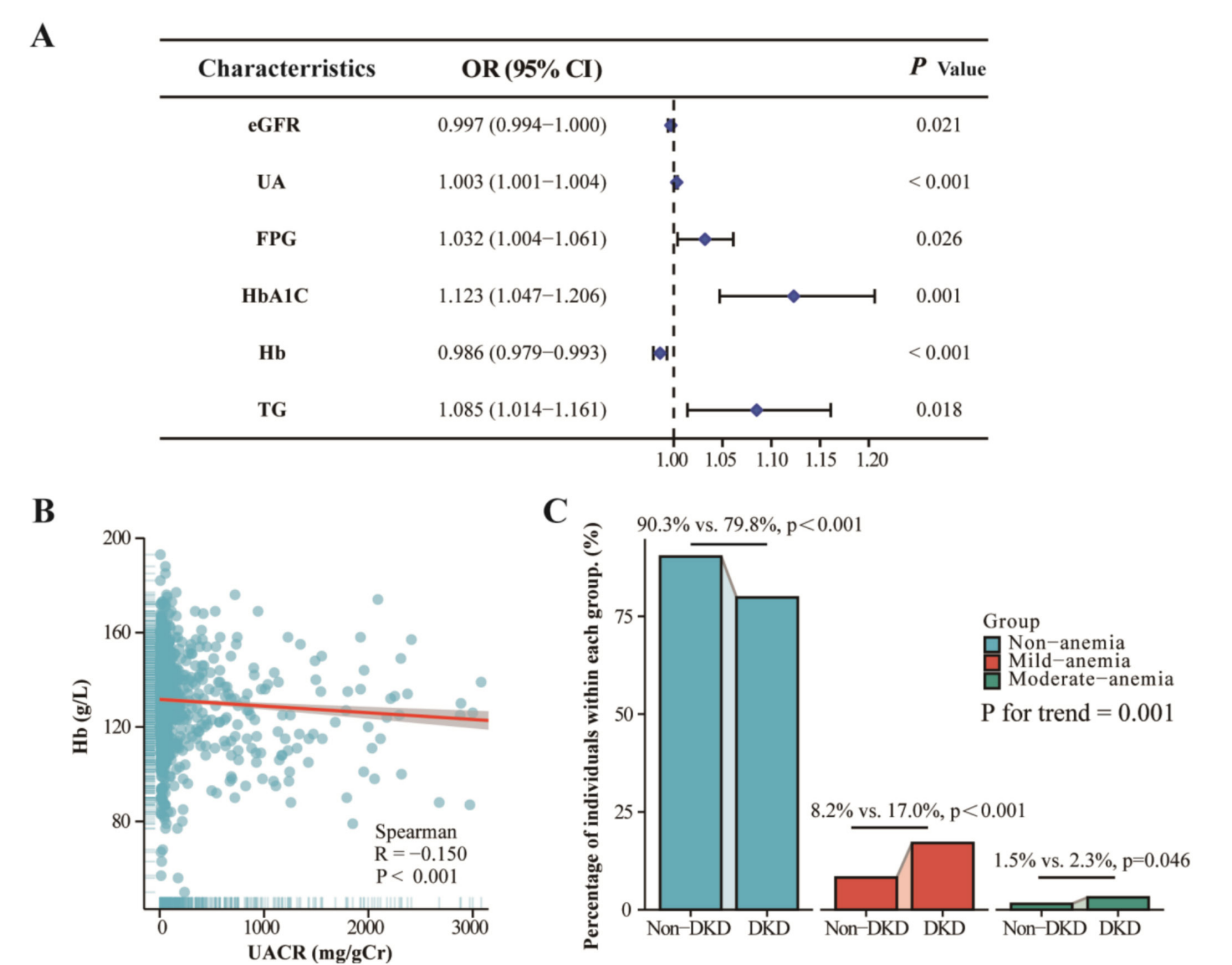
Hemoglobin concentration and the degree of anemia are related to the incidence of DKD. (A) Risk factors for DKD by logistic regression analysis; (B) Correlation between Hb levels and UACR by Spearman's correlation analysis; (C) Subgroup analysis, stratified by hemoglobin concentration.

**FIGURE 3:**
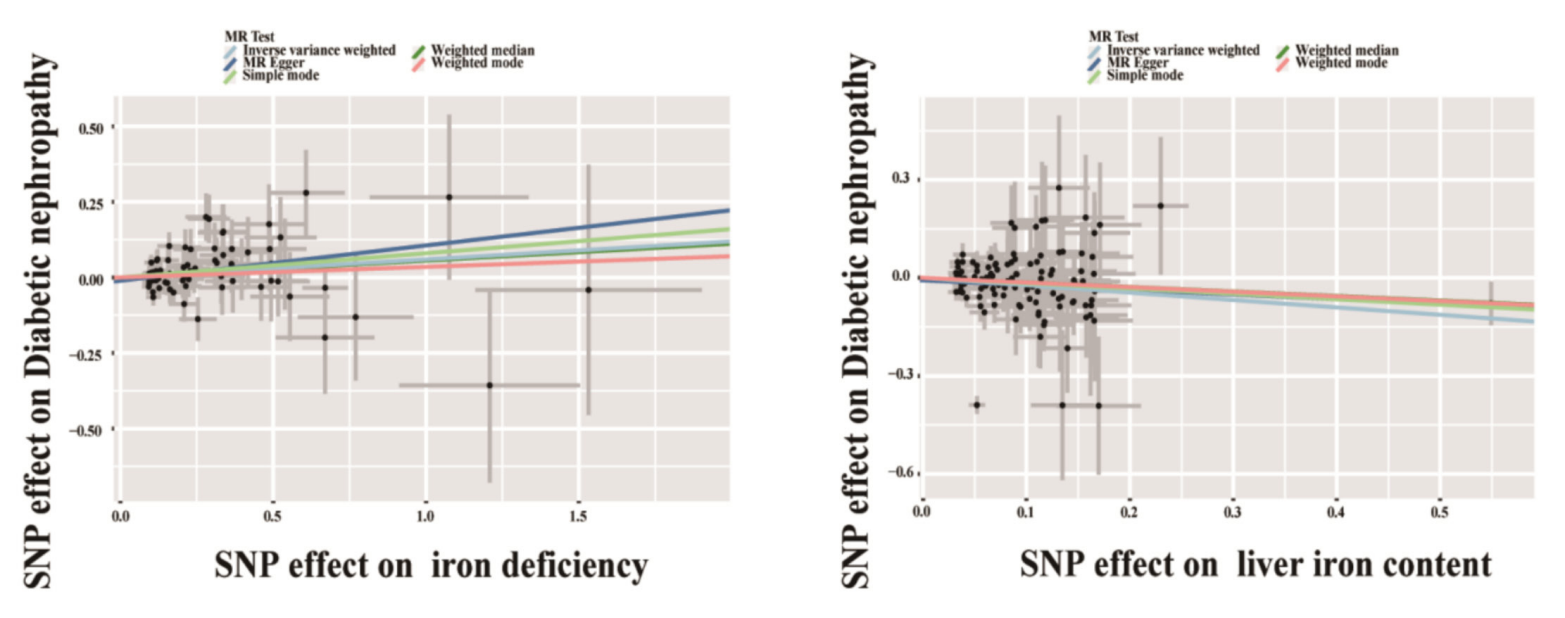
Scatter plots showing the causal effect of Iron deficiency or liver iron content on diabetic nephropathy (DN). Results are shown for different Mendelian randomization (MR) methods, including inverse variance weighted (IVW); MR‐Egger; simple mode; weighted median and weighted mode.

A multivariate logistic regression model was used to analyze the risk factors for DKD. After adjusting for all factors with a p‐value less than 0.05 in the univariate analysis, eGFR, UA, FPG, HbA1C, Hb, and TG were identified as significant independent risk factors for DKD. The ORs were 0.997, 1.003, 1.032, 1.123, 0.986, and 1.085, respectively (all *p* < 0.05) (Figure 2A). There was a negative correlation between Hb levels and UACR (*r* = −0.150, *p* < 0.001) (Figure 2B). The two groups of patients were stratified into three groups according to hemoglobin concentration: Non‐anemia (*N* = 604 in non‐DKD vs. *N* = 534 in DKD group, *p* < 0.001), mild‐anemia (*N* = 55 in non‐DKD versus *N* = 114 in DKD group, *p* < 0.001) and moderate‐anemia (*N* = 10 in mon‐DKD vs. *N* = 21 in DKD group, *p* = 0.046). With an increase in the degree of anemia, the proportion of individuals in the DKD group rises compared to the non‐DKD group (*p* for trend = 0.001) (Figure 3C).

### 3.3 Logistic Regression Analysis of the Impact of Hemoglobin on Occurrence of DKD

A high concentration of Hb was associated with an decreased prevalence of DKD (OR = 0.765; 95% confidence interval [95% CI], 0.650–0.900; *p* < 0.001), after adjusting for age, sex, BMI, HBA1C, FPG, ALP, UA, and TG levels but a lower fasting C‐peptide, TBIL, and eGFR. The association was maintained when the concentration of Hb were transformed into a categorical variable according to grading of anemia. Compared with individuals with non‐anemia, those with mild‐anemia and moderate‐anemia had an adjusted OR for DKD of 2.393(95% CI, 1.614–3.548; *p* < 0.001) and 2.948 (95% CI, 1.251–6.948; *p* < 0.001), respectively (*p* < 0.001) (Table 2). Given the higher prevalence of anemia among female patients, we included a gender‐stratified subgroup analysis in Table S1 to examine whether gender modulates the relationship between anemia and DKD. The findings revealed that in the male subgroup, significant statistical associations were observed across all parameters, including each SD increase in HB, anemia status, and anemia severity. In contrast, among females, only an SD increase in HB was associated with a reduced risk of DKD. These results suggest potential gender differences in the risk factors for DKD.

**TABLE 2:** Logistic regression analysis of the impact of hemoglobin on occurrence of DKD.

Characteristics	Univariate analysis	Model 1	Model 2
Hb (per‐SD increase)	0.833 (0.747–0.929)	0.754 (0.663–0.858)	0.765 (0.650–0.900)
Anemia (yes vs. no)	2.349 (1.709–3.229)	2.482 (1.795–3.432)	2.284 (1.544–3.378)
Grading of anemia			
No	Reference	Reference	Reference
Mild	2.344 (1.665–3.301)	2.466 (1.741–3.494)	2.393 (1.614–3.548)
Moderate	2.375 (1.109–5.089)	2.568 (1.192–5.534)	2.948 (1.251–6.948)
*p* for trend	< 0.001	< 0.001	< 0.001

*Note:* Model 1: Gender, Age, BMI. Model 2: Adjusted for Age, BMI, and all significantly different indicators in Table 1, excluding Hb and UACR.

### 3.4 MR Results for the Causal Effects of Iron‐Related Index on DKD Outcomes

A total of 82 SNPs were selected as IVs in the analysis of the causal effect of Iron deficiency on DKD. None of the horizontal pleiotropic outliers were found in the causal effect of Iron deficiency on DKD. In the MR analysis, the IVW method was used as the main approach to examine the causal effect (Table 3). Compared with the non‐iron‐deficient group, Iron deficiency was causally related with a 0.06‐fold increase in the risk of DKD (OR 1.06, 95% CI 1.003–1.12, *p* = 0.039). Genetic data involving iron‐related SNPs were employed to evaluate their influence on the development of DKD. The findings indicated that administering iron supplementation therapy is devoid of risks concerning susceptibility to DKD (*p* = 0.564). In order to evaluate the causal link between bodily iron reserves and DKD, we employed SNPs linked to liver iron content as IVs for examining their influence on DKD. None of the horizontal pleiotropic outliers were found in the causal effect of iron content on DKD. In the MR analysis, increased liver iron content is linked to a comparatively reduced risk of DKD occurrence (OR = 0.80, *p* = 0.020) (Table 3). The aforementioned indications imply that insufficient iron levels can contribute to the onset of DKD. Iron supplementation therapy is deemed safe, and the heightened accumulation of iron in the liver might function as a reservoir, potentially offering a preventative role against DKD. Furthermore, complementary MR approaches, including IVW, MR‐Egger; simple mode; weighted median and weighted mode, duplicated the previous findings with the same direction and magnitude of the causal effects (Figure 3). We also conducted an analysis of the impact of iron deficiency on other kidney outcomes (eGFR, proteinuria, CKD) and found no statistically significant differences, as shown in Table S2.

**TABLE 3:** Mendelian randomization results for the causal effects of Iron‐related index on DKD outcomes.

Factors	IVs in the MR study	OR (95% CI)	*p*
Iron deficiency	82	1.06 (1.003–1.12)	0.039
Iron supplements	84	4.66 (0.25–881.12)	0.564
Liver iron content	113	0.80 (0.66–0.97)	0.020

### 3.5 Underlying Mechanisms of the Impact of Iron Deficiency on the Kidneys by Bioinformatics Analysis

**FIGURE 4:**
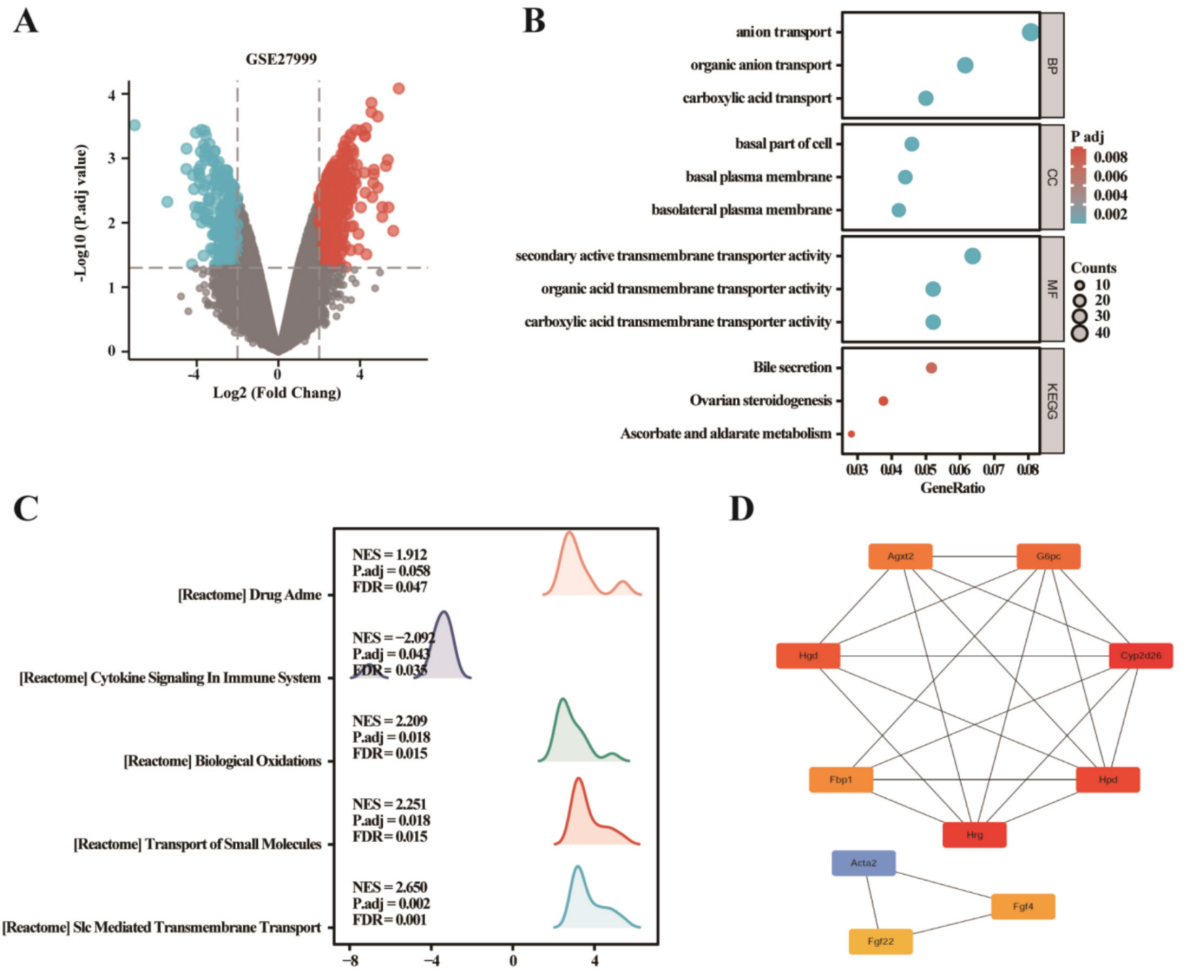
Underlying mechanisms of the impact of iron deficiency on the kidneys by bioinformatics analysis. (A) volcano plot; (B) GO/KEGG enrichment analysis; (C) GSEA enrichment; and (D) protein–protein interactions (PPI).

To identify DEGs linked with Iron deficiency in kidney, we downloaded relevant expression profiles from GSE27999. After consolidation and normalization of the RNA‐seq data, 580 DEGs were screened by the limma package (*p* < 0.05, |logFC| ≥ 2), as shown in the volcano plot (Figure 4A). GO/KEGG enrichment analysis was undertaken and displayed in Figure 4B. GSEA of the 580 DEGs revealed significant enrichment in pathways such as “Cytokine Signaling in the Immune System”; “Biological Oxidative”; “Transport of Small Molecules”; and “Slc Mediated Transmembrane Transport” (Figure 4C). Further analysis of protein–protein interactions (PPI) identified the top 10 hub genes: Cyp2d26, Hrg, Hpd, Hgd, G6pc, Agxt2, Fbp1, Fgf4, Fgf22, and Acta2 (Figure 4D).

## 4 Discussion

We performed a 1:1 PSM analysis on 1338 patients diagnosed with T2DM, uncovering a link between hemoglobin levels and susceptibility to DKD. Further stepwise logistic regression analysis indicated that both the presence and severity of IDA independently raised the risk of developing DKD. Since retrospective case–control studies can only establish associations, we employed a bidirectional MR approach, akin to a natural randomized controlled trial (RCT), utilizing publicly accessible large‐scale genome‐wide association study (GWAS) data. This approach allowed us to investigate the connection between IDA and the risk of DKD. Our results established a meaningful causal connection between IDA and the risk of DKD, and we found that iron supplementation had minimal impact on DKD. Using the GSE27999 dataset, we compared differences in kidney gene expression between individuals with iron‐deficient and normal diets. Gene Set Enrichment Analysis (GSEA) of the 580 genes that showed differential expression revealed significant enrichment in pathways such as “Cytokine Signaling in the Immune System,” “Biological Oxidative,” “Transport of Small Molecules,” and “Slc Mediated Transmembrane Transport.” Further analysis of PPI pinpointed the top 10 hub genes: Cyp2d26, Hrg, Hpd, Hgd, G6pc, Agxt2, Fbp1, Fgf4, Fgf22, and Acta2. These genes potentially play roles in the development of kidney issues related to iron deficiency. This study represents the initial connection between IDA and DKD, providing a basis for understanding the clinical mechanisms underlying DKD. It also sets the stage for assessing the effectiveness and safety of IDA treatment in diabetic patients for managing DKD.

Iron is a very important essential mineral for the cells in the body. Disordered iron metabolism can result in a range of chronic illnesses [21]. The equilibrium of iron within the body is intricately overseen through a meticulous coordination of distinct proteins engaged in uptake, excretion, and the storage/transport processes that occur within the cell [22]. The assimilation of iron takes place within the duodenum and is under the governance of the divalent metal transporter protein (DMT1) within intestinal cells. Iron enters the bloodstream via the iron transporter protein, subsequently binding with transferrin, and is then transported to the bone marrow and liver, facilitating the generation and retention of red blood cells (RBCs). Stored intracellular iron forms a bond with ferritin, a mechanism designed to avert cellular harm arising from the production of free radicals [23]. A surplus of iron coupled with instances of inflammation prompt the liver to generate hepcidin, which degrades ferroportin, thereby obstructing the absorption of iron into circulation and curbing its liberation from storage. Hepcidin synthesis wanes during episodes of hypoxia, escalated red blood cell production, and instances of iron deficiency [24]. Roughly 60% of T2DM patients fulfill the current criteria for iron deficiency [25]. Iron deficiency in people with diabetes is usually unrelated to insufficient dietary intake or gastrointestinal bleeding; instead, it stems from obstacles in the release of stored iron [26]. With cellular and systemic inflammation commonly exacerbated by obesity, elevated levels of inflammation‐induced hepcidin and ferritin play a crucial role in inducing functional iron deficiency among diabetic patients [27]. This phenomenon sheds light on why diabetes is a major contributor to anemia associated with chronic diseases. In our study, we also observed a higher prevalence of anemia in DKD. However, when assessing iron markers, serum ferritin levels, no significant difference was observed between the two groups, indicating the presence of functional iron deficiency.

As the pivotal excretory organ in the human body, the kidneys possess the capability to express various protein molecules related to iron metabolism. Through the processes of glomerular filtration, tubular reabsorption, and secretion, the kidneys assume a substantial role in governing the iron content as part of the body's iron homeostasis [28]. Iron that is initially bound to transferrin in the urine undergoes receptor‐mediated endocytosis, subsequently attaching to either TfR1 or cubilin [29]. This enables the PTC to reabsorb the iron. Once inside the cells, the absorbed iron can be employed in its reduced state, serving either for storage within ferritin or for utilization within mitochondria. Furthermore, iron can be reintroduced into circulation through the action of ferroportin [30]. Previous research suggests that relative hypoxia [31] and abnormal iron metabolism [32] are key mechanisms contributing to the development of DKD. IDA, a prevalent clinical condition, encompasses both of these pathological factors. However, the role of IDA in the onset of DKD remains unexplored. Our findings indicate that a reduction in hemoglobin levels in patients with type 2 diabetes is linked to the development of DKD. Moreover, both the presence and severity of IDA independently contribute to the risk of DKD. MR results also corroborate the potential of IDA to trigger DKD. The kidney, particularly the tubular tissue, undergo significant oxygen consumption due to their crucial reabsorption function. In diabetic patients, the early‐stage renal hyperfiltration and heightened metabolic consumption of substances like glucose create a relatively hypoxic stress environment [11]. If anemia coincides during this period, it can exacerbate the existing hypoxic condition, thereby intensifying oxidative stress and potential kidney damage. Previous studies have indicated that persistent iron deficiency combined with anemia can accelerate the advancement of cardiovascular conditions in both non‐diabetic and diabetic individuals [33]. The study conducted by Belma et al. demonstrates that effectively managing glycemia and anemia in diabetic patients leads to reductions in blood pressure, urine albumin secretion, and pulse rate.

Ferroptosis, is a form of programmed cell death triggered by iron overload. It is regulated by various aspects of iron metabolism, encompassing absorption, transport, storage, and utilization [34]. The body meticulously regulates iron levels to prevent cellular toxicity caused by iron overload, both at the cellular and systemic levels. Although extensive research has focused on ferroptosis in conditions such as acute kidney injury and renal cell carcinoma, its potential role in various chronic kidney diseases, including DKD, is gradually becoming evident [35, 36]. Iron overload and abnormal deposition of iron within the kidneys have been observed in various DKD animal models, with PTC being a particularly active site for iron ions and ROS. Studies indicate a close association between PTC injury under diabetic conditions and the activation of the ferroptosis pathway [10, 37]. Prior research has primarily concentrated on ferroptosis‐induced tissue or cellular injury due to iron overload, which seems contradictory to the outcome of our study on iron deficiency leading to DKD. However, our further analysis of changes in kidney mRNA expression following iron deficiency revealed activations in oxidative stress pathways and small molecule membrane transport pathways. In the context of diabetes, these changes resulting from iron deficiency could lead to increased renal reabsorption and transport burdens, potentially disrupting iron homeostasis within the kidneys and augmenting the unstable intracellular iron pool. This mechanism partially elucidates the potential link between IDA and DKD. For further insights into the key molecular players in this process, we conducted a protein–protein interaction analysis on differentially expressed genes (DEGs) and identified the top 10 critical genes: Cyp2d26, Hrg, Hpd, Hgd, G6pc, Agxt2, Fbp1, Fgf4, Fgf22, and Acta2. These genes could potentially serve as targets for comprehending the pathogenic mechanisms of DKD resulting from IDA. HRG‐7 mediates interorgan signaling between the intestine and extraintestinal tissues in *Caenorhabditis elegans* , functioning as a signaling factor under heme deficiency and potentially linked to human iron and heme metabolism regulation [38]. Sakine Sever reported a homozygous G6PC gene R83C missense mutation, leading to glycogen storage symptoms such as anemia and hyperuricemia [39]. In a Fah/Hpd double‐mutant mouse model, rapid tubular cell apoptosis and Fanconi syndrome were observed following hydroxyphenylpyruvate administration [40]. AGXT2 plays a pivotal role in kidney diseases, with its loss of function accelerating nephropathy progression [41]. HGF is critical in regulating the morphology and function of renal collecting ducts [42]. Acta2 modulates intracellular iron homeostasis and HIF‐1α via the β2‐M/HFE complex, ultimately inducing EMT in HK‐2 cells [43]. As previously mentioned, both iron overload and ferroptosis significantly contribute to the development of various chronic complications of diabetes, including DKD, raising concerns about iron supplementation in such populations. In our study, we utilized SNPs from individuals who received iron supplementation for IDA as IVs and employed MR analysis to ascertain that iron supplementation does not elevate the risk of DKD. Notably, the liver, a pivotal organ for iron storage and regulation, exhibited increased iron content, which acts as a protective factor for DKD. This observation suggests that the liver's iron storage pool could serve as a vital systemic iron buffer alongside hemoglobin. Consequently, our findings indicate that diabetic patients with coexisting IDA can safely undergo iron supplementation therapy without incurring additional DKD risk.

Our study indicates that within the diabetic context, having IDA is a contributing factor to the occurrence of DKD, and the use of iron supplementation therapy is both safe and feasible. However, our study does have some limitations: First and foremost, the lack of clinical follow‐up data makes it difficult to assess the relationship between IDA and the progression of DKD, as well as to examine how DKD improves before and after iron supplementation therapy. Moreover, even with normal eGFR levels, mild renal impairment may still influence anemia to some extent. However, the absence of EPO data limits our analysis, underscoring the need for further investigation in future studies. Second, while focusing on a single European population to explore causal relationships helps reduce biases due to population differences, its generalizability to other populations might be limited. The somewhat lenient selection of IVs associated with IDA could potentially lead to false‐positive results. Third, the presence of IDA or elevated liver iron levels does not definitively indicate whether the kidneys are experiencing an iron deficiency or overload. Further research is needed to establish animal models that mimic diabetes accompanied by IDA, enabling the assessment of changes in renal iron levels. Lastly, the bioinformatics analysis using the dataset from iron‐deficient diets is constrained by a small sample size and the inclusion of various components of the renal cortex, which could be influenced by elements like glomeruli or renal tubules. Therefore, future investigations should involve clinical studies that evaluate the risk of developing DKD in diabetic patients with IDA, both before and after treatment. Additionally, MR studies focused on iron metabolism and DKD risk are crucial within the Chinese population. Furthermore, more thorough exploration into creating and analyzing in vivo or in vitro models relevant to diabetes‐related iron deficiency is imperative.

## 5 Conclusion

IDA can elevate the risk of developing DKD. Kidney damage arising from iron deficiency may be connected to oxidative stress, heightened transport of small molecules, and additional factors. Administering iron supplementation as a remedy for IDA poses no risk concerning the occurrence of DKD, and an augmentation in liver iron content is linked to a diminished risk of DKD.

## Author Contributions

Huang Bin performed the data acquisition and drafted the work. The main statistical work was completed by Wen Wenjie. Ye Shandong and Huang Bin interpreted the patient data. Ye Shandong substantively revised it. All authors read and approved the final manuscript.

## Ethics Statement

The study protocol was approved by the ethics committee of the First Affiliated Hospital of USTC, Division of Life Science and Medicine, University of Science and Technology of China.

## Conflicts of Interest

The authors declare no conflicts of interest.

## Supporting information


**Table S1.** Logistic Regression Analysis of the Impact of Hemoglobin on Occurrence of DKD, Subgroup Analysis by Gender.
**Table S2.** Mendelian Randomization Results for the Causal Effects of Iron Deficiency on Kidney Outcomes.

## Data Availability

The datasets analyzed during the current study are available from the corresponding author.
