# Correction to “Iron‐Deficiency Anemia Elevates Risk of Diabetic Kidney Disease in Type 2 Diabetes Mellitus”

**DOI:** 10.1111/1753-0407.70174

**Published:** 2025-11-26

B. Huang, W. Wen, and S. Ye, “Iron‐Deficiency Anemia Elevates Risk of Diabetic Kidney Disease in Type 2 Diabetes Mellitus,” *Journal of Diabetes* 17, no. 2 (2025): e70060. https://doi.org/10.1111/1753‐0407.70060.

In the originally published version of this article, several inadvertent typographical errors were identified:

1. The total number of patients was incorrectly reported as 1398; the correct total is **1338** (669 patients in each group after 1:1 propensity score matching).
2. Accordingly, the total number of participants in the Abstract should also be corrected from 1398 to **1338**.
3. The reported male proportion should read **58.22%**.
4. The participants' age range should read **18–94 years** (instead of 19–84 years).
5. In Table 1, the number of female patients in the Non‐DKD group was wrongly listed as **386**; the correct value is **286**.

These corrections do not affect any statistical analyses or conclusions of the article.

We apologize for this error.

