# Supplementary material for: Iron‐Deficiency Anemia Elevates Risk of Diabetic Kidney Disease in Type 2 Diabetes Mellitus
Source: J Diabetes. 2025 Feb 19;17(2):e70060. doi: 10.1111/1753-0407.70060 (PMC11836615; doi:10.1111/1753-0407.70060)
Supplement: Supplementary file 1 — Table S1. Logistic Regression Analysis of the Impact of Hemoglobin on Occurrence of DKD, Subgroup Analysis by Gender. Table S2. Mendelian Randomization Results for the Causal Effects of Iron Deficiency on Kidney Outcomes. [file JDB-17-e70060-s001.docx]

**Supplementary Table 1.** Logistic Regression Analysis of the Impact of Hemoglobin on Occurrence of DKD，Subgroup Analysis by Gender

|  | Male | Female |
| --- | --- | --- |
| **Hb（Per-SD increase）** | **0.787(0.640-0.968)** | **0.751(0.568-0.994)** |
| **Anemia（Yes vs. No）** | **2.997(1.693-5.306)** | 1.611(0.914-2.840) |
| **Grading of anemia** |  |  |
| No | Reference | Reference |
| Mild | 2.501(0.556-11.252) | 1.522(0.817-2.835) |
| Moderate | **3.067(1.684-5.585)** | 2.025(0.613-6.694) |
| P for Trend | **< 0.001** | 0.092 |

Adjusted for Age, BMI, and all significantly different indicators in Table 1, excluding Hb and UACR.

**Supplementary Table 2.** Mendelian randomization results for the causal effects of Iron deficiency on kidney outcomes

| Outcomes | IVs in the MR study | OR (95%CI) | GWAS Data |
| --- | --- | --- | --- |
| eGFR | 30 | 1.00（0.99-1.02） | ieu-a-1284 |
| Proteinuria | 81 | 0.96 (0.82-1.12) | finn-b-R18_ISOLATED_PROTEINU |
| CKD | 58 | 0.98（0.93-1.03） | ebi-a-GCST008026 |
